# Surgical techniques to preserve continence after robot-assisted radical prostatectomy

**DOI:** 10.3389/fsurg.2023.1289765

**Published:** 2023-11-03

**Authors:** Stamatios Katsimperis, Patrick Juliebø-Jones, Anthony Ta, Zafer Tandogdu, Osama Al-Bermani, Themistoklis Bellos, Francesco Esperto, Senol Tonyali, Iraklis Mitsogiannis, Andreas Skolarikos, Ioannis Varkarakis, Bhaskar K. Somani, Lazaros Tzelves

**Affiliations:** ^1^ 2nd Department of Urology, Sismanoglio Hospital, National and Kapodistrian University of Athens, Athens, Greece; ^2^ Department of Urology, Haukeland University Hospital, Bergen, Norway; ^3^ Department of Urology, University College London Hospitals NHS Foundation Trust, London, United Kingdom; ^4^ Department of Urology, Campus Biomedico University of Rome, Rome, Italy; ^5^ Department of Urology, Istanbul Faculty of Medicine, Istanbul University, Istanbul, Türkiye; ^6^ Department of Urology, University of Hospital Southampton NHS Trust, Southampton, United Kingdom; ^7^ 2nd Department of Urology, National and Kapodistrian University of Athens, Athens, Greece

**Keywords:** prostate cancer, robot-assisted radical prostatectomy (RARP), continence recovery, preserving reconstruction techniques, functional outcomes

## Abstract

Radical prostatectomy significantly impacts the inherent anatomy of the male pelvis and the functional mechanisms of urinary continence. Incontinence has a considerable negative influence on the quality of life of patients, as well as their social and psychological wellbeing. Numerous surgical techniques have been demonstrated to support the preservation of continence during robot-assisted radical prostatectomy (RARP). In this in-depth analysis, we give a general summary of the surgical techniques used in RARP and their impact on incontinence rates.

## Introduction

Prostate cancer (PCa) is the second most common cancer among men (after skin cancer), with an estimated 1.4 million diagnoses worldwide in 2020 (1, 2). Robot-assisted radical prostatectomy (RARP) is considered one of the first-line treatment options for localized PCa. It is indubitably a challenging operation that has been refined through the years to achieve three main goals, namely, cancer treatment, preservation of urinary continence, and recovery of sexual function. These outcomes, referred to as trifecta, are of utmost importance for a patient (3). Apart from oncological efficacy, which is the most critical endpoint, urinary incontinence is a significant and long-term consequence that substantially decreases the quality of life (QoL) of patients (4).

While most men will remain continent at 12 months post-op (defined as no use of pads), early urinary continence rates vary with up to 70%–80% of men requiring the use of pads at 6 weeks and 20%–40% at 6 months and are, in turn, linked to low self-esteem and deterioration of psychological wellbeing (5–7). Multiple technical modifications have been proposed to improve urinary continence, such as bladder neck preservation (BNP) approaches (8), subapical urethral dissection (9), anterior and posterior reconstruction (10, 11), and nerve-sparing and Retzius-sparing (12). In this article, we review the available literature, summarizing the surgical techniques of RARP and their impact on incontinence rates.

### Surgical anatomy of the prostate

During RARP, the key goal is to leave the inherent anatomy of the male pelvis and the functional mechanisms of urinary continence undisrupted. The main anatomical landmarks are considered the detrusor apron, neurovascular bundles (NVBs), and Denonvilliers’ and endopelvic fascia.

**Figure 1:**
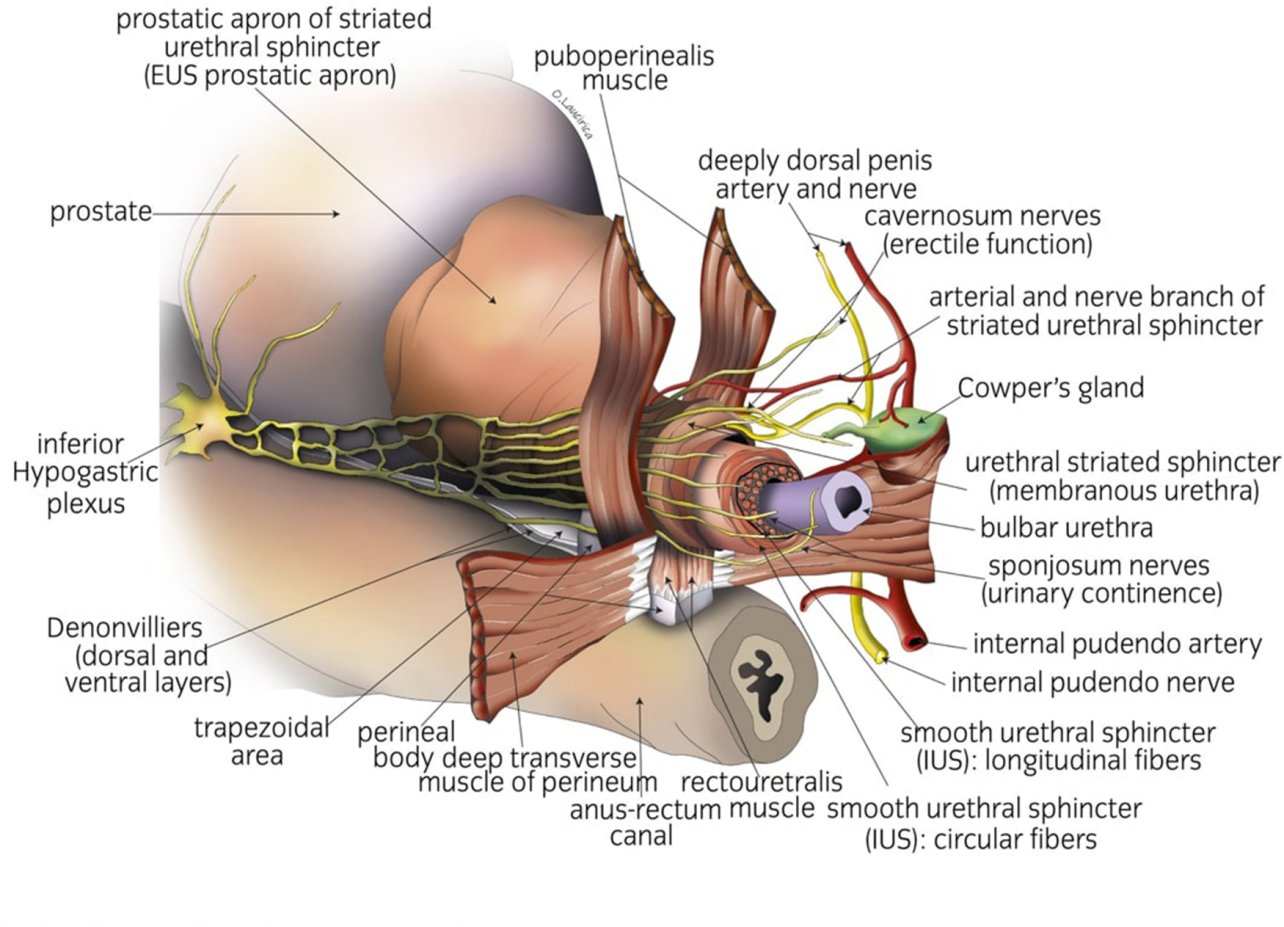
Anatomical landmarks to achieve early urinary continence.

For many years, there has been a common misconception that the bladder ends in front of the prostate. On the contrary, the bladder continues caudally in front of the prostate as an entity called detrusor apron, which is fixed to both the pubic bone and apex of the prostate. Puboprostatic ligaments are parts of the bladder apron (13). The detrusor apron is considered of major importance as it interconnects the two sphincteric mechanisms, namely, the vesical internal sphincter and external urethral sphincter, into one functional unit. The vesical internal sphincter, which is the circular part of the bladder continuing inside the prostate also covers the prostate from the outside (14). During bladder neck sparing, this musculature is stripped down until the bladder neck to help preserve as much bladder neck as possible. The external sphincter has two main parts. One is a circular horseshoe-shaped smooth muscle, responsible for continence preservation, and the other is an external striated muscle (14). The striated muscle ventrally overlaps the prostate way above the end of the apex. The lower boundary of the Santorini venous plexus is way under the anterior boundary of the striated sphincter (15). Knowledge of the anatomy helps preserve the external urethral sphincter during the control of the dorsal vein complex (DVC) (Figure 1).

In the past, the NVBs were considered two strains of nerves located in the posterolateral side of the prostate. Newer concepts in neural anatomy demonstrate that they are not two strains but a complete network of neurons interconnected from one side to the other. They form a surface at the level of Denonvilliers’ fascia (16). Denonvilliers’ fascia is one of the fascial components that surround the prostate gland, along with the prostatic capsule and lateral or endopelvic fascia. Like endopelvic fascia, Denonvilliers’ fascia is potentially not a single-layered structure but is composed of multiple sheets of tissues (17). This knowledge obtained from the advantage of magnification that laparoscopic surgery provided permitted the development of intra-, inter-, and extrafascial dissection during RARP. Avoiding the removal of Denonvilliers’ fascia during RARP is crucial for continence preservation. This tendinous structure continues from the base to the apex of the prostate and is considered to support the urethra and prostate as a fulcrum (18). The rest of Denonvilliers’ fascia across the posterior prostatic surface is considered to act as a hammock to support vesicourethral anastomosis (19).

### Surgical techniques to improve urinary continence rates

#### BNP approaches

Different approaches have been proposed to protect bladder neck circular fibers during RARP to achieve the preservation of urinary continence. Anterior, lateral, and anterolateral dissection planes are most commonly utilized. Regardless of the chosen technique, protecting the bladder neck as high as possible has been found to preserve urinary continence. Deliveliotis et al. (20) described the first reported cases of BNP that resulted in improved continence rates in patients who underwent open radical prostatectomy. Freire et al. were the first to describe a technique of BNP in RARP (21). In their series of 347 patients who had undergone the BNP technique vs. 271 patients who had undergone a standard RARP, they reported significantly better continence rates at 4 and 12 months with BNP (65.6% vs. 26.6% at 4 months; 86.4% vs. 81.4% at 12 months) (21). Hashimoto et al. performed a retrospective multivariate analysis on predictors of continence in patients undergoing RARP with BNP and found that BNP was significantly associated with early continence (22). In a relatively recent systematic review and meta-analysis, BNP was associated with significantly better urinary continence outcomes at 3–4 months compared with patients who underwent RARP without BNP [odds ratio (OR), 2.88; 95% confidence interval (CI), 1.52–5.48; *p* = 0.001], 12 months (OR, 2.03; 95% CI, 1.10–3.74; *p* = 0.02), and 24 months (OR, 3.23; 95% CI, 1.13–9.20; *p* = 0.03) after RARP (23). The risk of increased positive surgical margin (PSM) still remains controversial regarding BNP. In the former meta-analysis, there was no difference in the rate of overall PSM (OR, 1.00; 95% CI, 0.72–1.39; *p* = 0.99) and that of PSM at the prostate base (OR, 0.49; 95% CI, 0.21–1.13; *p* = 0.09) between the two groups. A newer described technique of extended bladder neck sparing is complete urethral preservation, during which the intraprostatic urethra is preserved in cases with no central zone tumors. During this technique, the bladder neck is not dissected until the level of the verumontanum, where the urethra is usually thinner and essentially permits a urethra–urethral instead of a vesicourethral anastomosis. Initial oncological and functional outcomes are very encouraging, with reported immediate continence rates of nearly 50% after removing the catheter (24).

#### Neurovascular bundle preservation

When NVB techniques were first adopted by surgeons, their main goal was to preserve erectile function. Through the years, a better understanding of the anatomical localization of the prostatic nerves has led some urologists to theorize that damage to the NVB might affect the continence mechanism. For instance, the cavernosal nerves of the NVB have been shown to directly innervate the membranous urethra. On the other hand, some surgeons contend that it is the meticulous dissection during nerve-sparing rather than the NVB itself that is responsible for improved outcomes of urinary continence (25, 26). Regardless of the real reason behind this, NVB preservation seems to be strongly associated with improved continence recovery after RARP. Reeves et al. conducted a systematic review and meta-analysis involving 13,749 patients and showed that NVB sparing compared with non-NVB sparing resulted in improved early urinary continence rates up to 6 months postoperatively (27). Park et al. (28) demonstrated similar results. In their study, 84.6% of the patients treated with nerve-sparing RARP were continent at 12 months compared with 74.6% of those having non-nerve-sparing RARP. Nerve-sparing was also significantly associated with recovery of urinary continence on multivariate analysis (hazard ratio, 0.713; 95% CI, 0.548–0.929; *p* = 0.012).

Nerve-sparing techniques are categorized, based on fascial dissection, into intrafascial, interfascial, and extrafascial. The working plane in the intrafascial dissection is between the prostatic capsule and the several layers of periprostatic fascia. It allows total NVB preservation but with a greater risk for PSM. In the interfascial dissection, the working plane is between the prostatic fascia and the lateral pelvic fascia and medial to the NVB. The prostatic fascia is retained intact, which allows a greater safety margin decreasing the PSM. In the extrafascial approach, the dissection is carried over the prerectal fat and the endopelvic fascia. It is important to plan the level of dissection based on the preoperative multiparametric MRI and biopsy, to allow for more accurate local staging.

The classic nerve-sparing RARP technique involves the dissection of NVB from the posterolateral arc between the prostate and Denonvilliers’ fascia. This technique has been further refined, leading to the development of newer techniques. Such a technique is the so-called Veil of Aphrodite (29), where the initial plane of dissection is between the prostatic fascia and lateral pelvic fascia from the base of the seminal vesicles. The interfascial dissection then proceeds between the 1 and 5 o’clock positions for the right side and between the 6 and 11 o’clock positions for the left side, leaving the detached prostatic fascia as a supportive structure. Kaul et al. (29) reported that 29% of patients who underwent RARP with Veil of Aphrodite were continent at the time of catheter removal, 97% were continent at the 12-month follow-up, and the median time to continence was 14 days, demonstrating an advantage in regaining early continence. Ghani et al. then modified this technique, extending the interfascial dissection more anteriorly between the 11 and 1 o’clock positions (30). The idea behind this procedure called super Veil is that 25% of the NVBs can be found on the anterior surface of the prostate. Due to its greater complexity, this procedure is usually preserved for low-risk patients. Galfano et al. presented another modified nerve-sparing technique in which NVBs are preserved by releasing them retrogradely (31). In his technique, after reaching the space of Retzius, the anterior neck of the bladder is dissected without entering the endopelvic fascia or ligating the DVC. The vas deferens and seminal vesicles are then dissected through an incision made in the posterior neck of the bladder. Using this technique, the NVB can be released easily from below, achieving a good avascular plane between the prostatic fascia and NVB. The main goal is to connect the space created by separating the NVB from the anterior prostate surface with the previously created Denonvilliers’ space. The presented results were very promising as continence was reached immediately in 85.9% of the patients and 98.4% were continent at 1 year. However, these results were deeply questioned by experts in the field (32). In 2017, Cochetti et al. (33) presented another novel neurovascular sparing technique called the PERUSIA technique (PERUSIA stands for Posterior, Extraperitoneal, Robotic, Under Santorini, Intrafascial, Anterograde). In their technique, after inducing pneumo-Retzius, they follow an anterograde–intrafascial dissection approach in a lateral manner with enlargement of the retroprostatic space toward the prostatic pedicles. Following the medial aspect of the Veil of Aphrodite they reach the anterior periprostatic tissue and detouch it bluntly from the fascia, without damaging the accessory neurovascular plate. This technique has proved its feasibility and efficacy, with reported continence rates of 69% the day after the removal of the catheter, 92% at 3 months, and 97% at 12 months after surgery (34).

#### Subapical urethral dissection and preservation of the external sphincter and membranous urethral length

As we have previously mentioned, a big part of the external sphincter is placed inside the prostate between the apex and the verumontanum (35, 36). Due to the anatomical variations of the shape of the apex, a considerable part of the sphincter is covered by apical tissue (37–39). For that reason, preserving the full functional length of the urethra also helps preserve part of the external sphincter. Mungovan et al. demonstrated that each extra millimeter of urethral length, which was measured preoperatively via MRI, was associated with early continence recovery (40). These findings were also justified by Song et al., who showed that the preoperative and postoperative maximum urethral length was significantly associated with urinary continence at 6 and 12 months after RARP (41). Michl et al. demonstrated that careful dissection of the apex had a beneficial effect on early and long-term urinary continence rates compared with a wide excision (26). In a recent retrospective study by Hoeh et al., implementing full functional-length urethral sphincter and NVB preservation in patients undergoing RARP resulted in improved long-term (12 months) continence rates (defined as no pad or one pad) of 91% (42).

#### Preservation of supporting anatomical structures: Retzius-sparing and hood technique

In 2010, Galfano et al. described Retzius-sparing RARP, a posterior approach to the prostate via access through the Douglas space (12). In Retzius-sparing RARP, a transverse incision is first made at the peritoneal reflection underlying the rectovesical pouch. The vas deferens and seminal vesicles are then recognized and mobilized. Antegrade dissection begins at the posterior and posterolateral surfaces of the prostate, and the NVBs are swept laterally. The bladder neck is divided, and the DVC is released with sharp dissection. The urethra is cut below the apex, and the prostate is freed. This approach preserves all the anatomical structures anterior to the prostate such as the DVC, pubovesical and puboprostatic ligaments, detrusor apron, and endopelvic fascia, providing anterior bladder support and leading to better continence rates. Galfano et al. demonstrated immediate continence in >90% of the patients. Numerous later publications (43–45) supported these findings. One common critic for this technique is that the benefit of continence does not exist after 6 months when continence rates equalize with those of the standard approach. However, in a systematic review and meta-analysis published in 2020, higher continence recovery was seen up to 12 months (46). Another major concern regarding Retzius-sparing RARP is that existing studies have consistently reported higher PSM rates (47). In the MASTER study, a systematic review and meta-analysis of four randomized controlled trials (RCTs) and six prospective observational studies, PSM rates in ≤pT2 tumors were statistically significantly higher following Retzius-sparing RARP as compared with standard RARP (47). In another study coming from Japan, the authors demonstrated that Retzius-sparing RARP is associated with higher PSM rate in anterior tumors, but not in posterior tumors, compared to conventional RARP (48). The preservation of Santorini plexus and detrusor apron probably makes the distance between the tumor edge and the resection plan a lot smaller, which, in turn, affects PSM. The steep learning curve involved to achieve optimal outcomes is also worthy of mention when talking about Retzius-sparing RARP (49, 50).

In 2021, Tewari et al. demonstrated their own RARP technique, preserving periurethral anatomical structures in the space of Retzius and sparing the pouch of Douglas, which they called the hood technique (51). The contents in the space of Retzius are preserved anteriorly, and the preserved tissue after prostate removal has the appearance of a “hood” comprising the detrusor apron, arcus tendineus, puboprostatic ligament, anterior vessels, and some fibers of the detrusor muscle. This hood surrounds and safeguards the membranous urethra, external sphincter, and supportive structures. Among patients receiving the “hood technique,” the continence rate exceeded 80% at 4 weeks following catheter removal. By 48 weeks post-catheter, the continence rate rose to 95%. The technique also had a low rate of PSM (6%).

### Reconstructive techniques to improve urinary continence recovery

#### Posterior reconstruction (Rocco stitch)

In 2001, Rocco et al. first presented their technique of posterior reconstruction in open retropubic prostatectomy, aiming to achieve improved continence recovery (52). During posterior reconstruction, the surgeon sutures the remaining Denonvilliers’ fascia to the posterior aspect of the rhabdosphincter and the posterior median raphe. Then, the posterior layer of the rhabdosphincter is sutured to the posterior surface of the bladder. This transfers the urethral sphincter cranially, lessens the stress in the anastomosis, and also gives the bladder neck pelvic support. Bearing these in mind, preserving Denonvilliers’ fascia seems to be of utmost importance for the success of this technique. In 2007, Rocco et al. adapted their technique to RARP, demonstrating significantly shortened time to continence recovery and feasibility of the technique laparoscopicaly (53). Since its introduction, other surgeons have used and slightly modified the Rocco stich. Rocco et al. tried to synthesize the evidence in a systematic review, showing improved continence recovery at 30 days postoperatively (54). In a more recent review, Rosenberg et al. demonstrated that posterior reconstruction in RARP may result in better continence 1 week after removal of the catheter compared with RARP without reconstruction (although it is also possible that it is no better). However, it may make little to no difference at either 3 or 12 months after surgery (55).

#### Anterior reconstruction (Patel stich)

Similar to what Walsh first described in open retropubic prostatectomy, Patel suggested his technique of anterior reconstruction in RARP (56, 57). After ligating the DVC, Patel placed a periurethral retropubic stitch to the pubic bone in a figure of eight pattern, providing suspension to the rhabdosphincter. The suspension technique resulted in significantly greater continence rates at 3 months after RARP compared with the group without the Patel stich (92.8% vs. 83%, *p* = 0.013).

#### Combined anterior and posterior reconstruction

Urologists mostly preferred using combined anterior and posterior reconstruction or referred by many as total reconstruction as it has shown better results regarding continence rates. In the first RCT comparing RARP with total reconstruction (group A) to standard RARP (group B), Koliakos et al. showed improved continence recovery (58). At 7 weeks, the continence rates were 65% and 33% for groups A and B, respectively. In two more RCTs that followed, Hurtes et al. and Student et al. presented similar results (59, 60). In 2019, Porpiglia et al. presented a large series of >1,000 procedures of RARP with total reconstruction showing excellent results in the early recovery of urinary continence with 79.66% of the patients being continent at 3 months after catheter removal (61). Furthermore, a systematic review by Checcucci et al. showed that total reconstruction facilitates a faster and higher continence recovery compared with the standard approach or posterior reconstruction or anterior reconstruction only (62).

#### Newer concepts: single port transvesical robotic radical prostatectomy

In 2021, Kaouk et al. demonstrated a totally different approach in RARP utilizing the new da Vinci single port surgical system (63). In their technique, after placing the patient in a supine position, a suprapubic incision, two fingerbreadths above the pubic symphysis, is made. The bladder is then identified, and the new da Vinci SP access port is used for robot docking. The bladder is insufflated to 12 mmHg pressure, and the robot is docked.

The operation starts with the incision of the posterior bladder neck in a semilunar fashion, extending to 5 and 7 o’clock, respectively (64). The dissection is proceeded posteriorly to reach the vasa deferentia and seminal vesicles bilaterally. After transecting the vas deferens, Denonvilliers’ fascia is incised, and the posterior plane is developed between the prostate and the rectum. Next, the incision of the bladder neck is completed anteriorly to reach the endopelvic fascia. The urethra is divided distal to the apex of the prostate, preserving a long urethral stump (64). Prior to urethrovesical anastomosis, a posterior reconstruction is performed. With their technique, Kaouk et al. have reported excellent continence rates. The median time using a Foley catheter after surgery was 4 days, 56% of the patients had immediate continence after Foley removal, and the continence rate was 96.7% at 3 months postoperatively (64). Even though more studies are needed, this approach seems very promising.

## Conclusions

RARP is a procedure that has undergone numerous modifications to improve patient outcomes without compromising oncologic safety. In this narrative review, we tried to present the current perspectives and recent advancements in surgical techniques regarding continence preservation. The comprehensive comparison of various techniques has been significantly hampered by the lack of a standardized method for reporting results and the scarcity of RCTs. As our understanding of the complex periprostatic anatomy expands, it becomes obvious that the surgeon’s experience is of utmost importance to decide the optimal surgical approach. Therefore, attention should be focused on conducting randomized trials, which are essential when comparing novel techniques and can assist surgeons on optimizing their outcomes.
